# Optical Coherence Tomography- and Optical Coherence Tomography Angiography-Based Evaluation in Treatment-Naïve Non-Exudative Macular Neovascularization

**DOI:** 10.3390/jcm14186375

**Published:** 2025-09-10

**Authors:** Geun Young Moon, Jong Seok Park, Ki Woong Bae

**Affiliations:** 1 Department of Ophthalmology, Nowon Eulji University Hospital, Seoul 01830, Republic of Koreapjs4106@eulji.ac.kr (J.S.P.); 2 Department of Ophthalmology, Eulji University College of Medicine, Daejeon 34824, Republic of Korea

**Keywords:** age-related macular degeneration (AMD), non-exudative macular neovascularization, optical coherence tomography (OCT), optical coherence tomography angiography (OCTA)

## Abstract

**Background/Objectives:** We evaluated the clinical features and natural course of treatment-naïve non-exudative macular neovascularization (NE MNV) associated with age-related macular degeneration in Korean patients. **Methods:** This retrospective longitudinal study of 21 eyes of 21 patients with NE MNV involved a chart review of best corrected visual acuity (BCVA), optical coherence tomography (OCT), and OCT angiography parameters. **Results:** This study included 13 men (13/21, 61.9%) and 8 women (8/21, 38.1%), with a mean age of 71.5 ± 9.1 years. The average follow-up period was 15.1 ± 11.8 (range 6.0–49.6) months, and 14 eyes (66.7%) demonstrated exudative changes on OCT scans. The baseline BCVA was 0.15 ± 0.18 logMAR. The initial central macular thickness (CMT), subfoveal choroidal thickness, and the outer retinal layer thickness were 265.3 ± 37.1, 245.2 ± 95.2, and 86.6 ± 5.3 μm, respectively. Cox proportional hazards analysis revealed that older age (hazard ratio [HR]: 1.096, 95% confidence interval [CI]: 1.002–1.200; *p* = 0.045), larger baseline CMT (HR: 1.025, 95% CI: 1.002–1.049; *p* = 0.035), and larger baseline MNV (HR: 1.618, 95% CI: 1.035–2.529; *p* = 0.035) were significant risk factors for exudative changes. **Conclusions:** We observed the clinical features and natural course of NE MNV in Korean patients and identified that significant risk factors for exudative changes in NE MNV included old age, initially thick CMT, and larger MNV size at baseline. For eyes with NE MNV that have risk factors of exudative conversion, more frequent observation is recommended to ensure the appropriate management.

## 1. Introduction

Age-related macular degeneration (AMD) is one of the most common causes of blindness worldwide, with an estimated prevalence of 288 million by 2040 [1]. Neovascular AMD (nAMD) is characterized by retinal vascular leakage and fluid accumulation that are associated with macular neovascularization (MNV), which is a major sight-threatening complication of AMD [2].

Currently, intravitreal injections of anti-vascular endothelial growth factor (VEGF) agents are the gold standard for the treatment of nAMD [3,4,5,6]. Delay in commencing treatment in patients with newly diagnosed nAMD is one of the most important factors that is associated with poor visual outcome [7]. Therefore, early detection and treatment are crucial to improving the vision of patients with nAMD.

The introduction of optical coherence tomography angiography (OCTA) has facilitated the detection of subclinical MNVs [8,9,10], and different nomenclatures have been introduced for these lesions, including “quiescent,” “subclinical,” and “non-exudative” [11]. In 2013, Querques et al. [12] first conducted research that focused on non-exudative (NE) MNVs, which is characterized by the absence of active exudation on optical coherence tomography (OCT) scans in patients with AMD who are frequently asymptomatic. Several subsequent studies have explored the incidence, prevalence, and natural history of NE MNVs [8,9,13,14,15,16,17,18]. Recent studies have identified several biomarkers for predicting the exudative conversion of NE MNV [9,12,15,16,17,18,19]. Bae et al. [20], in a recent study, conducted in Korea using OCTA reported that the risk of exudation from NE MNV was 22.5% at 12 months and specific OCTA biomarkers, including anastomosis and loops and vessel density for disease progression. Furthermore, a recent systemic review [16] showed that exudative progression occurred in 20.9% at the 1 year and several risk factors, such as large MNV area, rapid growth of MNV, and pigment epithelium detachment (PED) growth during the follow up.

It is well established that the clinical features and subtypes of AMD differ between Asian and Western populations [21]. However, NE MNV is a relatively recent concept introduced with the advent of OCTA, and its ethnic or regional variations remain unclear owing to limited research. Most studies related to NE MNV involved a small number of patients and relatively short follow-up periods. Furthermore, reports for Asian population are rare, which highlights the importance of our study on Korean patients with NE MNV.

Therefore, this study aimed to provide further insight into the identification of the potential risk factors for exudative conversion in treatment-naïve NE MNVs, as well as to investigate the clinical course and natural history of NE MNV.

## 2. Materials and Methods

### 2.1. Patients

This retrospective longitudinal study was approved by the Institutional Review Board (IRB) of the Nowon Eulji University Hospital (IRB no. 2023-10-005-001; approval date 3 November 2023) and adhered to the tenets of the Declaration of Helsinki. The requirement for informed consent was waived by the same IRB because of the retrospective nature of this study and the analysis of anonymized clinical data. We consecutively included patients with treatment-naïve NE MNV who visited the Retina Clinic of Nowon Eulji University Hospital between January 2019 and September 2023. During the study period, all patients with AMD who visited the clinic were consecutively enrolled and screened to determine whether they met the NE MNV diagnostic criteria. Furthermore, patients aged >50 years who were followed up for at least 6 months were enrolled in this study. Typically, follow-up visits were typically scheduled every 3 months; however, the intervals were occasionally adjusted based on the patient conditions or at the physician’s discretion.

NE MNV was diagnosed using multimodal imaging and presented as ill-defined hyperfluorescent lesions without leakage on fluorescein angiography (FA), hyperfluorescent plaques in the late phase of indocyanine green angiography (ICGA), and neovascular networks identified on OCTA [12,22]. On structural OCT, NE MNVs are defined as an irregular elevation of the retinal pigment epithelium (RPE) and are not accompanied by subretinal/intraretinal fluid accumulation, regardless of the patient’s visual symptoms [12,22]. During follow-up, exudative progression from NE MNV was considered when exudative changes, including subretinal fluid and/or intraretinal fluid or subretinal hyperreflective materials, were present on OCT scans [15].

Medical records, including BCVA, spectral domain (SD) OCT, and OCTA parameters, were obtained and analyzed. The exclusion criteria were as follows: (1) concomitant other retinal or optic nerve diseases, including diabetic retinopathy, diabetic macular edema, epiretinal membrane, macular hole, retinal vascular occlusion, glaucoma, and uveitis; (2) history of intravitreal injections (including anti-VEGF drugs and/or steroids in the study eye), or laser photocoagulation; (3) history of intraocular surgery, except cataract extraction; (4) cases of inappropriate SD OCT or OCTA images (owing to severe media opacity, low signal strength, incomplete ocular examinations, or segmentation errors); Specifically, images were eligible in this study for OCT scans with Q-value > 25 and OCTA scans >30, whereas those scoring ≤20 were either scans that were repeated or excluded from the study [23,24]; and (5) extensive refractive errors exceeding ±4 diopters.

### 2.2. Ophthalmic Examinations

All patients underwent a complete ophthalmic examination, including best corrected visual acuity (BCVA) assessment, refractive error measurement, intraocular pressure measurement, slit-lamp examination, and dilated fundus examination. Ocular laboratory examinations included color fundus photography (TRC-NW8; Topcon, Oakland, CA, USA) and ultra-widefield retinal imaging (Optos Daytona P200T; Optos PLC, Dunfermline, UK). OCTA and en face structural OCT images were acquired using Spectralis OCT (Heidelberg Engineering, Heidelberg, Germany). FA and ICGA were performed using a confocal scanning laser ophthalmoscope (Spectralis HRA; Heidelberg Engineering, Heidelberg, Germany).

All participants underwent SD-OCT at each visit. A macular thickness map was obtained using 31 horizontal raster B-scans (1024 A scans per line) covering a 30° × 30° area centered on the fovea. The central macular thickness (CMT) was recorded in a circle with 1 mm diameter on the Early Treatment Diabetic Retinopathy Study thickness map using the built-in Spectralis OCT software (version 6.16.7). Furthermore, we obtained the outer retinal layer (ORL) in the circle with 1 mm diameter on the ETDRS thickness map using an automatic segmentation process with a built-in OCT program. The ORL is situated between Bruch’s membrane (BM) and the outer limiting membrane, which includes the space between the BM and RPE, the RPE itself, and the outer segments of the photoreceptors. This allows the targeted analysis of all structures that are mostly affected by MNV [17]. The subfoveal choroidal thickness (SCT), which is the perpendicular distance from the BM to the scleral–choroidal junction at the subfovea, was measured using a caliper that was included with the OCT software. In all patients, we performed OCTA in a 3 mm × 3 mm scanning area centered on the fovea and selected an avascular slab to identify the MNV.

### 2.3. Data Analysis

A junior retinal specialist (K.W.B.) and a senior resident (G.Y.M.) blinded to the clinical information independently performed manual measurements of the MNV area and retinal thickness parameters. Any discrepancies were adjudicated by a senior retinal specialist (J.S.P.), and measurements from the two graders were averaged for statistical analysis. To measure the MNV area, en face images were exported and analyzed using ImageJ version 1.50 (National Institutes of Health, Bethesda, MD, USA; available at http://imagej.net/ij/index.html). Two trained graders (KWB and GYM) independently outlined the MNV area using a polygon selection tool. The size of the MNV was measured in square millimeters by utilizing a scale that was based on the pixel distance and the known distance in millimeters for each scan dimension [15]. The average measurement of the MNV area from both grades was used for statistical analysis. The intraclass correlation coefficients (ICC) were used to evaluate the agreement between the individual measurements obtained by both readers, and the clinical and demographic characteristics of the study cohort were analyzed using descriptive statistics and a commercially available software package (SPSS Statistics 25.0; IBM, Armonk, NY, USA). The means and standard deviations are presented for continuous variables, and the frequencies and percentages are used for categorical variables. After the normality of the data was evaluated, parametric tests were used for normally distributed data and non-parametric tests were used for data that did not have a normal distribution. The significance of the differences between variables was evaluated using the Mann–Whitney *U* or Wilcoxon signed-rank test.

Based on the presence of exudative changes during the follow-up period, the study population was divided into two groups, with one group exhibiting exudation (activated MNV group) and the other without exudation (quiescent group). The Kaplan–Meier method was used to estimate the cumulative incidence of exudation development, and Kaplan–Meier curves were generated using SPSS Statistics 25.0 (IBM, Armonk, NY, USA). Scatter plots were constructed using Prism 5 (GraphPad Software, San Diego, CA, USA). The Cox proportional hazard model was used to determine the risk factors and calculate the hazard ratios associated with the progression to exudative changes. Statistical significance was set at *p* < 0.05.

## 3. Results

**Figure 1:**
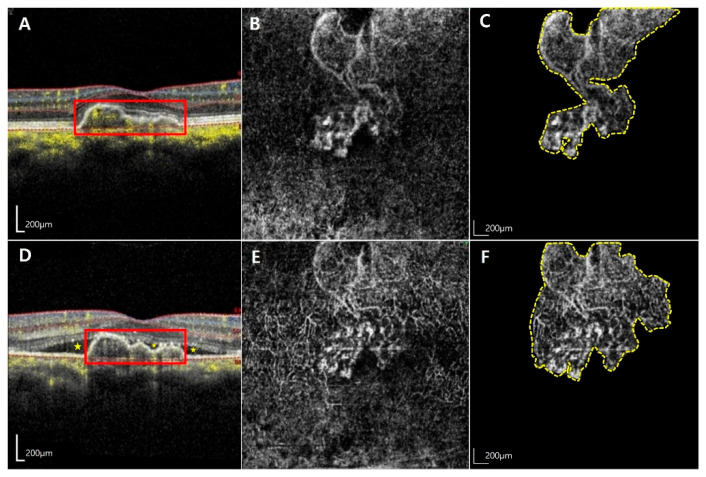
**Representative images from a 65-year-old man with increased macular neovascularization (MNV) size and exudative changes in untreated non-exudative (NE) MNV.** The images are from a 65-year-old man who was diagnosed with activated MNV. The first row displays the optical coherence tomography (OCT) and optical coherence tomography angiography (OCTA) images at the initial visit (**A**–**C**), where the best corrected visual acuity (BCVA) was 1.0 decimal scale, and flat pigment epithelial detachment (red-outlined rectangle) was noted (**A**). The initial MNV (yellow-dotted polygon) area was measured at 1.96 mm^2^ (**C**). The second row shows the OCT and OCTA images at the follow-up visit 7 months later (**D**,**E**), and BCVA decreased to the 0.7 decimal scale. At this visit, OCT scan (**D**) confirmed the presence of exudative change (yellow asterisk), and OCTA revealed a significant increase in the MNV (yellow-dotted polygon) area to 3.32 mm^2^ within a short period (**F**).

**Figure 2:**
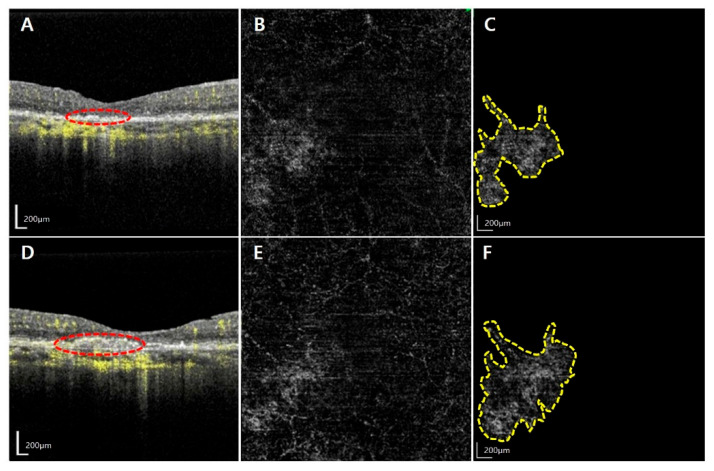
**Representative images from an 80-year-old woman with non-exudative macular neovascularization (NE MNV) without exudative changes.** The images are from an 80-year-old woman in the quiescent MNV group. The first row shows the optical coherence tomography (OCT) and optical coherence tomography angiography (OCTA) scans at this time (**A**–**C**). At the initial visit, her best corrected visual acuity was 0.7 decimal scale, and OCT image (**A**) revealed the presence of flat pigment epithelial detachment (red-dotted circle), with an initial MNV (yellow-dotted polygon) area of 0.74 mm^2^ (**C**). The patient underwent regular check-ups for 2 years, and vision was maintained. OCT performed at the final visit indicated no signs of exudative change (**D**), and the size of the MNV (yellow dotted polygon) was 0.98 mm^2^ presented at the second row (**E**,**F**).

Among a total of 972 patients with AMD, 21 eyes from 21 patients were diagnosed with NE MNV and were accordingly included in this study. The mean age of the participants was 71.5 ± 9.1 years. Among the study population, 8 were females (38.1%) while 13 were males (61.9%). The activated MNV group and quiescent group comprised a higher proportion of males (11/21, 78.6%) and females (5/8, 71.4%), respectively. However, this difference was not statistically significant (*p* = 0.081). Representative cases of the activated MNV group and the quiescent group are presented in Figure 1 and Figure 2.

The average follow-up period was 15.1 ± 11.8 (range: 6.0–49.6) months and 14 eyes (66.7%) showed the exudative change at OCT scans during the observation. For all the study eyes, the BCVA was 0.15 ± 0.18 logMAR. The CMT, SCT, and ORL thickness at the first visit were 265.3 ± 37.1, 245.2 ± 95.2, and 86.6 ± 5.3 μm, respectively. Baseline CMT was 278.9 ± 31.2 and 238.1 ± 32.9 μm for the activated MNV group and quiescent group, respectively, wherein former had a significantly thicker CMT than the latter (*p* = 0.020). However, there were no significant differences in the other baseline characteristics. The average baseline BCVA of the activated MNV group and the quiescent group were 0.18 ± 0.21 and 0.09 ± 0.06 logMAR, respectively, and the mean BCVA at the final visit were 0.22 ± 0.23 and 0.08 ± 0.10 logMAR, respectively. Though the activated MNV group appeared to have worse visual acuity at baseline than the quiescent group, the difference was not statistically significant (*p* = 0.689). There was no significant intergroup difference in the fellow eye status (*p* = 0.529). The average baseline MNV size was 1.48 ± 1.42 and 0.62 ± 0.58 mm^2^ and the mean MNV size at the final visit was 2.00 ± 1.40 and 0.80 ± 0.80 mm^2^ in the activated MNV group and quiescent group, respectively. Detailed demographics and baseline characteristics of the study population are presented in Table 1.

Among the 14 eyes that exhibited exudative changes during follow-up, the ORL thickness significantly increased from 87.6 ± 6.1 to 106.4 ± 22.2 μm (*p* = 0.012), and the SCT increased from 237.1 ± 98.9 to 264.7 ± 110.8 μm (*p* = 0.013), compared to the baseline. The average duration from baseline to the onset of exudative changes was 13.4 ± 10.7 (range: 1.6–31.9) months.

In the case of seven eyes that did not show any exudative changes during follow-up, there were no significant differences in the ORL thickness, CMT, and SCT. However, there were significant intergroup differences between the activated MNV group and quiescent group in the initial CMT, CMT changes, and changes in the ORL thickness (*p* < 0.05). Furthermore, there was no significant intergroup difference in the initial ORL thickness, which indicated that although the ORL thickness increased continuously in the activated MNV group, there was minimal change in the quiescent group (Table 2). The interobserver agreement between two graders (K.W.B. and G.Y.M.) in the measurements of the SCT and MNV size was consistent, with high ICC values (0.966 [0.933–0.983] and 0.944 [0.889–0.972], respectively).

**Figure 3:**
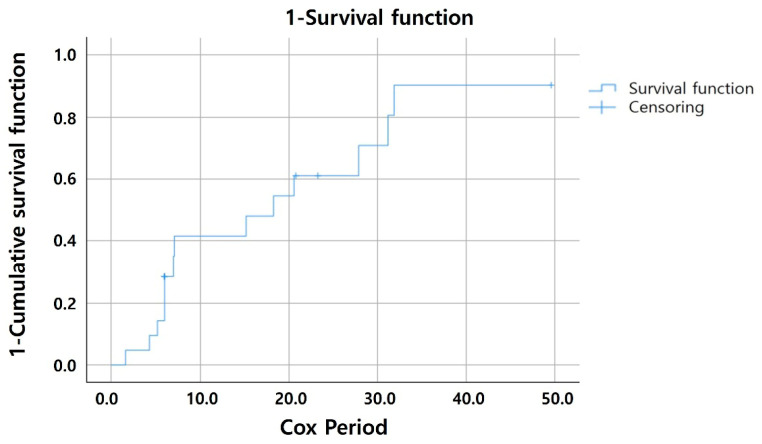
**Kaplan–Meier Curve of Exudative Change Occurrence.** This figure illustrates the timing of exudative changes in patients over a 24-month follow-up period, as analyzed using a Kaplan–Meier curve. It was observed that 57.1% of patients exhibited exudative changes by the 12-month mark, and which increased to 80.9% by 24 months.

Considering the different follow-up durations among patients, the Cox proportional hazard model was used to assess the risk factors for exudative changes in patients with NE MNV. Older age at baseline (hazard ratio [HR] = 1.096, 95% confidence interval [CI]: 1.002–1.200; *p* = 0.045), greater CMT at baseline (HR = 1.025, 95% CI: 1.002–1.049; *p* = 0.035, and larger MNV size at baseline (HR = 1.618, 95% CI: 1.035–2.529; *p* = 0.035) were identified as risk factors for exudation. Furthermore, the timing of exudative changes was analyzed using the Kaplan–Meier curve. Exudative changes occurred in 57.1% and 80.9% of patients by 12 and 24 months, respectively (Figure 3).

**Figure 4:**
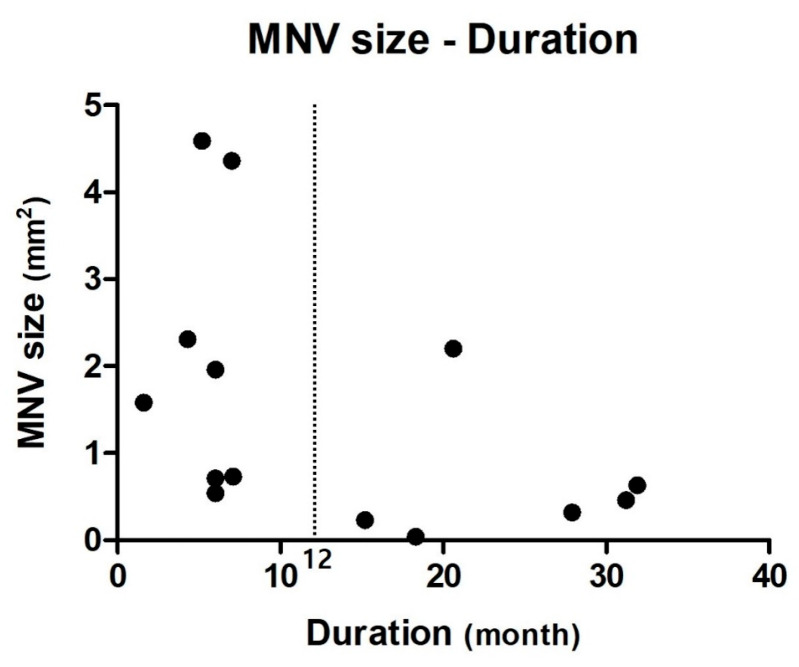
**Associations between the duration and macular neovascularization (MNV) size at baseline.** Scatter plot showing the relationship between the size of MNV at baseline and duration in the activated MNV group. Based on the scatter plot, the eyes were categorized into the acute and late activation groups, with a 12-month cut-off duration (black dashed line). The acute and late groups comprised eight and six eyes, respectively. The acute activation group showed a significantly shorter duration of exudative conversion (5.4 ± 1.8 months) compared to the late activation group (24.2 ± 7.1 months) (*p* = 0.001). The initial MNV size was significantly larger in the acute activation group (2.10 ± 1.60 mm^2^) compared to the late activation group (0.65 ± 0.79 mm^2^) (*p* = 0.020).

After establishing a cutoff period of 12 months from the initial visit to the onset of exudative changes, the activated MNV Group was subdivided into two subgroups (Figure 4). The acute activation group (<12-month duration until the onset of exudative changes) consisted of eight eyes, whereas the late activation group (>12-month duration until the onset of exudative changes) included six eyes. The duration of progression to wet AMD showed a significant intergroup difference, with values of 5.4 ± 1.8 and 24.2 ± 7.1 months, respectively (*p* = 0.001). The initial MNV size was 2.10 ± 1.60 and 0.65 ± 0.79 mm^2^ in the acute activation and late activation groups, respectively, which revealed a statistically significant intergroup difference (*p* = 0.020).

## 4. Discussion

This study evaluated the natural history of 21 NE MNVs in 21 Korean patients. During the follow up, 14 eyes (66.7%) showed exudative changes, and the mean duration between the initial visit and the onset of exudative change was 13.4 ± 10.7 (range: 1.6–31.9) months. A total of 21 patients with AMD were followed for an average duration of 15.1 ± 11.8 (range: 6.0–49.6) months. Significant intergroup differences were observed between the activated MNV group and quiescent group in OCT parameters, including the initial CMT, CMT change, and change in ORL thickness. According to Invernizzi et al. [17], the thickening of the ORL that begins in the area where the exudative MNV will develop long before the exudation appears and accelerates significantly in the last 2 months before the event may precede type 1 MNV activation. Moreover, those authors suggested that structural changes in the outer retina, including swelling of the RPE and the outer segments of photoreceptors as well as changes in the BM, may contribute to ORL thickening. In our study, the thicker ORL in the activated MNV group might be close to an exudative change. The Cox proportional hazards model was used to identify the risk factors for exudative change in NE MNVs and these included older age, initial thicker CMT, and larger MNV size at baseline, which were identified as significant predictors for disease progression.

Hanutsaha et al. [25] first reported the detection of treatment-naïve NE MNV in ICGA in 1998. However, ICGA is not routinely used to identify NE MNV because of the invasive nature of the examination, the risk of an allergic reaction, and the time-consuming testing process. Recently, with the increasing availability of OCTA, studies have begun to evaluate the characteristics of NE MNVs. Several previous reports on NE MNVs have reported variable exudation rates of 20% to 80%, owing to differences in the study design, patient numbers, the severity and duration of AMD, and follow up periods [26].

According to a recently conducted meta-analysis [16], the exudative progression rates of 20.9% and 30.7% at 1 year and 2 years, respectively. In our study, 66.7% of patients showed exudative changes, which was much higher compared to previous reports. This discrepancy may be attributable to differences in the study design and population. Querques et al. [15] presented the disease entity of ‘quiescent’ MNV as a type 1 MNV based on the absence of exudative change on OCT scans for at least 6 months. The cut-off threshold of the 6-month duration without exudation was applied to exclude the presence of a pre-clinical stage of an ordinary exudative type 1 MNV from the group of dormant NE MNV, and cases that showed activation within 6 months were referred as the short-term activation group. In our study, with the exclusion of patients who showed activation within 6 months, the exudation rate at 2 years slightly decreases to 41.7%. In a recent study by Cho et al. [18], the authors excluded subjects who experienced exudation within the first 6 months. As a result, the conversion rates in this report were 9.8% at 1 year and 21.3% at 2 years, which are relatively low compared with those of previous studies.

Several studies have reported the factors associated with the development of exudation in NE MNVs [26]. Eyes with a large MNV at baseline were at a higher risk of exudation, as reported by Fukushima et al. [27], Teo et al. [28] and Cho et al. [18]. Our study also confirmed that an initially large MNV is a significant risk factor for exudative changes. We presented a scatter plot showing the period until exudative progression and MNV size in Figure 4. The activated MNV group was arbitrarily divided into two subgroups based on a cut-off threshold of 12-month duration: the acute activation group exhibited exudation within 12 months, whereas the late activation group had a >12-month duration at exudation; moreover, the former group had significantly larger MNV than the latter. This suggests that patients with large MNV may be at a high risk of early exudative progression, and close and frequent monitoring is necessary to detect disease activation.

Serra et al. [29] and Solecki et al. [19] found that an increase in the height of PED was associated with exudation. In our study, the thickness of the ORL, which is the area that contains the PED, was significantly increased in the activated MNV group compared with that in the quiescent group. In addition to the previously identified factors, older age was associated with a risk of exudation of NE MNV, and this result is consistent with that of a recent study by Cho et al. [18].

Our study had some limitations. First, the study had a retrospective design. Second, the follow-up period was relatively short, and the sample size was small. Moreover, the different follow-up durations among patients, which is a limitation that is inherent in real-world data and our retrospective study design, could have introduced bias. To account for this, we employed the Cox proportional hazards model to effectively control for time-dependent variables when assessing the risk of exudative changes. Moreover, the MNV size was manually measured from OCTA images, in contrast to other OCT parameters that were obtained automatically. This may generate concerns regarding the proper measurement of MNV size. However, two independent researchers evaluated the extent of MNV separately and a high ICC was obtained, which attest to the reliability and reproducibility of the results. Among patients in the quiescent group, some MNVs could have been activated in the future, which may constitute a source of a bias. Lastly, we did not conduct a detailed qualitative analysis of MNV. Many MNVs were too small to evaluate the vascular patterns and features.

## 5. Conclusions

This retrospective longitudinal study characterized the clinical presentation and natural progression of NE MNV in Korean patients. A substantial proportion of eyes with NE MNV developed exudative features during the follow-up period, and this emphasizes the need for careful observation. Cox proportional hazards analysis identified older age, increased baseline CMT, and larger baseline MNV size as significant predictors of exudative conversion. These results are in agreement with previous reports and enhance our understanding of the clinical course and natural history of NE MNV. Future investigations that incorporate prolonged follow-up and larger populations would necessitate for the management and treating patients with this condition.

## Figures and Tables

**Table 1:** Demographics and clinical characteristics of eyes with MNV at baseline.

Factors	Total (n = 21)	Activated MNV Group (n = 14)	Quiescent Group (n = 7)	*p*-Value
Age, years	71.5 ± 9.1	72.1 ± 8.3	70.3 ± 9.9	0.799
Sex, female (%)/male (%)	8 (38.1)/13 (61.9)	3 (21.4)/11 (78.6)	5 (71.4)/2 (28.6)	0.081
Follow-up period, months	15.1 ± 11.8	14.2 ± 9.7	16.8 ± 15.1	0.443
Baseline BCVA, logMAR	0.15 ± 0.18	0.18 ± 0.21	0.09 ± 0.06	0.689
Baseline CMT, μm	265.3 ± 37.1	278.9 ± 31.2	238.1 ± 32.9	0.020 *
Baseline SCT, μm	245.2 ± 95.2	237.1 ± 98.9	261.5 ± 84.9	0.535
Baseline ORLT, μm	86.6 ± 5.3	87.6 ± 6.1	84.7 ± 1.8	0.360
AMD status of the fellow eyes (%)				
Dry AMD	6 (28.6)	3 (21.4)	3 (42.9)	
Neovascular AMD	5 (23.8)	4 (33.3)	1 (14.3)	0.529
Geographic atrophy	2 (9.5)	2 (14.3)	0 (0.0)	
MNV size at baseline, mm^2^	1.19 ± 1.27	1.48 ± 1.42	0.62 ± 0.58	0.149

MNV, macular neovascularization; BCVA, best corrected visual acuity; logMAR, logarithm of the minimum angle of resolution; CMT, central macular thickness; SCT, subfoveal choroidal thickness; ORLT, outer retinal layer thickness; AMD, age-related macular degeneration. Statistically significant results (*p * <  0.05) are denoted by an asterisk.

**Table 2:** Intergroup comparison of the functional and anatomical changes during the follow-up.

Factors	Activated MNV Group (n = 14)		*p*-Value	Quiescent Group (n = 7)		*p*-Value
	Baseline	Progression		Baseline	Last Visit	
BCVA, logMAR	0.18 ± 0.21	0.22 ± 0.23	0.463	0.09 ± 0.06	0.08 ± 0.10	0.686
CMT, μm	278.9 ± 31.17	342.9 ± 111.3	0.087	238.1 ± 32.9	236.9 ± 37.0	0.606
SCT, μm	237.1 ± 98.9	264.7 ± 110.8	0.013 *	261.5 ± 84.9	283.9 ± 93.3	0.128
ORLT, μm	87.6 ± 6.1	106.4 ± 22.2	0.012 *	84.7 ± 1.8	85.4 ± 3.9	0.546
MNV size, mm^2^	1.48 ± 1.42	2.00 ± 1.40	0.012 *	0.62 ± 0.58	0.80 ± 0.80	0.017 *

MNV, macular neovascularization; BCVA, best-corrected visual acuity; logMAR, logarithm of the minimum angle of resolution; CMT, central macular thickness; SCT, subfoveal choroidal thickness; ORLT, outer retinal layer thickness. Statistically significant results (*p* < 0.05) are denoted by an asterisk.

## Data Availability

The datasets are not available for public access because of patient privacy concerns. However, they are available from the corresponding author upon reasonable request.
